# Through the professional’s eyes: transfers of care during pregnancy, childbirth and the postpartum period

**DOI:** 10.1186/s12913-020-4941-0

**Published:** 2020-02-11

**Authors:** Cherelle M. V. van Stenus, Max B. Poorthuis, Magda M. Boere-Boonekamp, Ariana Need

**Affiliations:** 1 Departments of Public Administration, and Health Technology and Services Research, University of Twente, P.O. Box 217, 7500 AE Enschede, The Netherlands; 2 Department of Change Management & Organizational Behaviour, University of Twente, P.O. Box 217, 7500 AE Enschede, The Netherlands; 3 Department of Health Technology and Services Research, University of Twente, P.O. Box 217, 7500 AE Enschede, The Netherlands; 4 Department of Public Administration, and Twente Graduate School, University of Twente, P.O. Box 217, 7500 AE Enschede, The Netherlands

## Abstract

**Background:**

In the Netherlands, the obstetric and neonatal healthcare system consists of multiple healthcare organizations. Due to this system, transfers between healthcare professionals are inevitable. Transfers can interrupt the continuity of care, which is an important aspect of care quality. The aim of this study is to examine how healthcare professionals transfer their clients and to understand factors that facilitate or impede continuity of care.

**Methods:**

We conducted 15 semi-structured interviews with community midwives (4), obstetricians/clinical midwives (4), maternity care assistants (4), and youth healthcare nurses (3) between June and September 2016. After discussing the meaning of transfers of care, we introduced a vignette on the care process of a pregnant woman and asked about the methods the professional would use to transfer a client and about factors that facilitate or impede continuity of care.

**Results:**

Obstetric and neonatal healthcare professionals mentioned 19 factors that facilitate or impede continuity of care. The facilitating factors were, e.g., usage of protocols and standard formats, transfers in person, being accessible, and multidisciplinary meetings. Impeding factors included, e.g., acute situations, experienced hierarchy, insufficient knowledge of protocols, and privacy concerns.

**Conclusion:**

Professionals mentioned a broad variety of factors facilitating and impeding continuity of care.

## Background

The healthcare system that provides care during pregnancy, childbirth and the postpartum period in the Netherlands is somewhat different from healthcare systems in other countries [1]. The obstetric care system consists of three levels of care between which pregnant women can be transferred, depending on their risk level [2]. Primary care is provided by community midwives, who guide pregnant women with a low-risk pregnancy from the beginning of their pregnancy until the postpartum period [3].

If the pregnancy and childbirth proceed without complications, women and their partners can choose where to give birth [4]. Childbirth can take place at home, at the outpatient department in the hospital, or at a birth hotel [5]. Community midwives will guide all low-risk childbirths. If complications arise (or if the risk of complications increases), a community midwife transfers women to the secondary care level, where they are guided by obstetricians and/or clinical midwives [6, 7]. Severely ill women or women with a severely ill unborn child are transferred to a tertiary care level, which is a care facility with highly specialized care [8]. Women with an increased risk level always give birth at a hospital.

Criteria for transfer between primary and secondary care levels are listed in the “List of Obstetric Indications” [5]. After giving birth, Dutch mothers receive maternity care at home for at least a week. During childbirth in a primary care setting, the maternity care assistant supports the midwife. After childbirth, the maternity care assistant guides the new parents and support them to take care of the newborn [9]. Following maternity care, the care of the newborn child and the parents is transferred to the youth healthcare [10].

The youth healthcare contributes to children (0–18 years) growing up healthy and safe in the Netherlands. Their core tasks are: 1) monitoring the physical, social, psychological, and cognitive development of children, 2) assessing the social, pedagogical, and physical environment of children and the family they grow up with, 3) identifying problems and specific disorders timeously, and 4) giving preventative information, vaccinations, advice, instructions, and guidance to children and parents individually or in groups, while focusing on empowering the parents and the child [11].

The obstetric and neonatal healthcare system in the Netherlands is complex, due to clearly demarcated care levels with corresponding healthcare professionals and a large number of healthcare facilities. A consequence of the Dutch system is that all women will most likely experience several transfers during their pregnancy, childbirth, and the postpartum period, because of the process described above [12]. In this article, we look at how these transfers of care can influence the continuity of care of the Dutch obstetric and neonatal healthcare system from a healthcare professional perspective.

Continuity of care, an important aspect of care quality, can be interrupted by transfers of care [13, 14]. In general, interrupted continuity of care is noticeable to clients when they experience gaps in their care provision, which will occur when the coordination between healthcare professionals and between healthcare professionals and the client is inadequate [15]. Price and Lau explored professional perspectives on continuity of care in a healthcare system [15]. An extended model of continuity of care, derived from the continuity of care model Haggerty and Reid portrayed, was presented with four elements of a healthcare system that influence continuity: 1) Circle of care, which Price and Lau defined as “an individual patient’s healthcare system,” in which there is system continuity between the healthcare professionals involved with a single client; 2) Environmental factors that indirectly influence the circle of care and continuity; 3) Provider connectedness that enables continuing care between professionals; 4) Communication patterns that support continuity between professionals and between professionals and their clients. Price and Lau formulated 10 communication patterns which they then combined as one element [15, 16].

Several studies showed that transfers of care can negatively influence clients’ experiences with obstetric and neonatal healthcare [17–21], especially when the transfers take place during labor or childbirth [8, 18, 20, 22]. Feelings of losing self-control, being fearful of what will happen and unfulfilling expectations were negative outcomes of transfers of care concerning clients’ psychological well-being [18, 20, 22]. On the other hand, experiences from healthcare professionals with continuity of care are underexposed [23]. The research question addressed in this paper is therefore: What are healthcare professionals’ perceptions of the relevant factors that influence the continuity of care in the Dutch obstetric and neonatal healthcare system?

## Methods

### Study design

We conducted a vignette study combined with exploratory interviews with 15 obstetric and neonatal healthcare professionals who work on all three levels of care between which pregnant women can be transferred from June to September 2016. These professionals all work at an obstetric or neonatal healthcare organization covering one of the eastern provinces (Overijssel) in the Netherlands. According to the criteria of the Dutch Medical Research Involving Human Subjects Act, this study did not need to be submitted for ethical approval by a Medical Ethics Committee [24]. The study was reviewed and approved by the institutional ethics committee on April 19, 2018 (reference number 18384).

### Procedure

For the interviews, we approached professionals who were actively involved in the process of providing obstetric and neonatal healthcare and transferring women during their pregnancy, childbirth, and/or the postpartum period. This recruiting strategy can be classified as purposive sampling. After consultation with medical professionals, it was estimated that a number of 15 professionals would be sufficient to answer the research questions. When executing the research, this estimation proved to be correct as in the final interviews no more new themes emerged and there was a high rate of duplication of responses. To be eligible for participation, professionals needed to (1) be involved in the process of transferring clients to other obstetric and neonatal healthcare professionals, or (2) work in the province Overijssel in the eastern part of the Netherlands. Professionals were selected purposefully, based on their profession and the organization they work for.

We first approached four community midwife practices – two were located in a rural area (clients who mainly live in villages or on the countryside) and two in an urban area (clients who mainly live in bigger cities). We then invited secondary healthcare professionals who work at the maternity ward from two different hospitals to participate in our study. We also contacted regional care managers from one large and one smaller organization that provide maternity care during childbirth and in the first week thereafter, requesting whether they wanted to participate in our research. We sought contact with two youth healthcare organizations, and phoned and emailed potential participants. If they were interested in participating, we sent an information letter and made an appointment for the interview.

### Participants

The study population consisted of 15 professionals. From the four midwife practices, four community midwives participated in the study. Two of them worked in small midwife practices (one or two midwives), while the other two worked in larger midwife practices (4 to 10 midwives). From the secondary healthcare professionals, a resident obstetrician, two clinical midwives, and a nurse participated. The regional care managers from the maternity care organizations provided us with four maternity care assistants who were prepared to share their experiences. Three youth healthcare nurses from two youth healthcare organizations, responsible for preventive healthcare in the first four postpartum weeks, also agreed to an interview. All but one of the participants were women with an average of 10 years’ experience as obstetric and neonatal healthcare professionals. Table 1 presents a summary of the participants’ characteristics.

**Table 1:** Characteristics of research participants

	Participants
	*N* = 15
Profession	
Community midwife	4
Obstetrician/clinical midwife	4
Maternity care assistants	4
Youth healthcare nurses	3
Gender	
Male professionals	1
Female professionals	14
Average age	42.3 years
Average years of experience	9.8 years

### Interview protocol

We used a semi-structured interview protocol (see the interview protocol in Additional file 1). The interview protocol was read by consulted medical professionals who were not otherwise involved in the study. Each interview consisted of three parts. In the first part, participants were asked personal questions such as age and how many years they have worked for the organization. The protocol included the topic ‘transfer of care’ to make sure that interviewer and interviewee used the same definition.

**Fig. 1:**
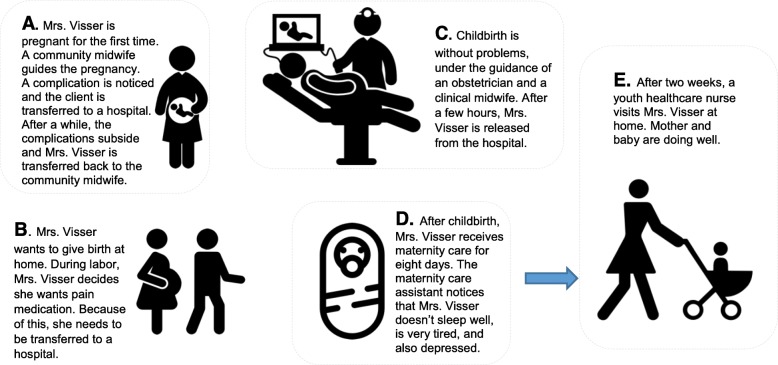
Vignette interview protocol

In the second part, the interviewer introduced a vignette (Fig. 1) [25]. During the interview, the participants were asked to imagine the proposed vignette as their client. The vignette was the same for every participant, but the process of care was different for every profession, depending on their role in the care chain. Participants described which acts they would perform to transfer the client, how they would contact other professionals and how they would receive feedback from the professional who continued providing the care. In the final part of the interview, we asked targeted questions about how professionals experienced transfers of care.

### Procedures

We conducted 14 interviews (with 15 participants) between June and September 2016. The interviews had an average length of 58 min (in a range from 38 to 81 min). The first author conducted 13 interviews, while an assistant researcher performed the 14th interview. Interviews were recorded, transcribed verbatim and anonymized immediately afterwards. The first author and assistant researcher made field notes of important statements in case of unclear recordings. There were 13 individual interviews, whereas two maternity care nurses participated simultaneously in the other one. The statements in this interview were transcribed separately. This was possible because the respondents answered the questions in turn. Their voices on the recordings could also be clearly distinguished. The statements from this interview are henceforth seen two separate interviews. Ten interviews were carried out face to face at the organizations where the participants worked. In two cases, the participants offered to come to the researcher’s organization. There were also three Skype interviews. Participants gave verbal consent to record the interviews.

### Data analysis

**Fig. 2:**
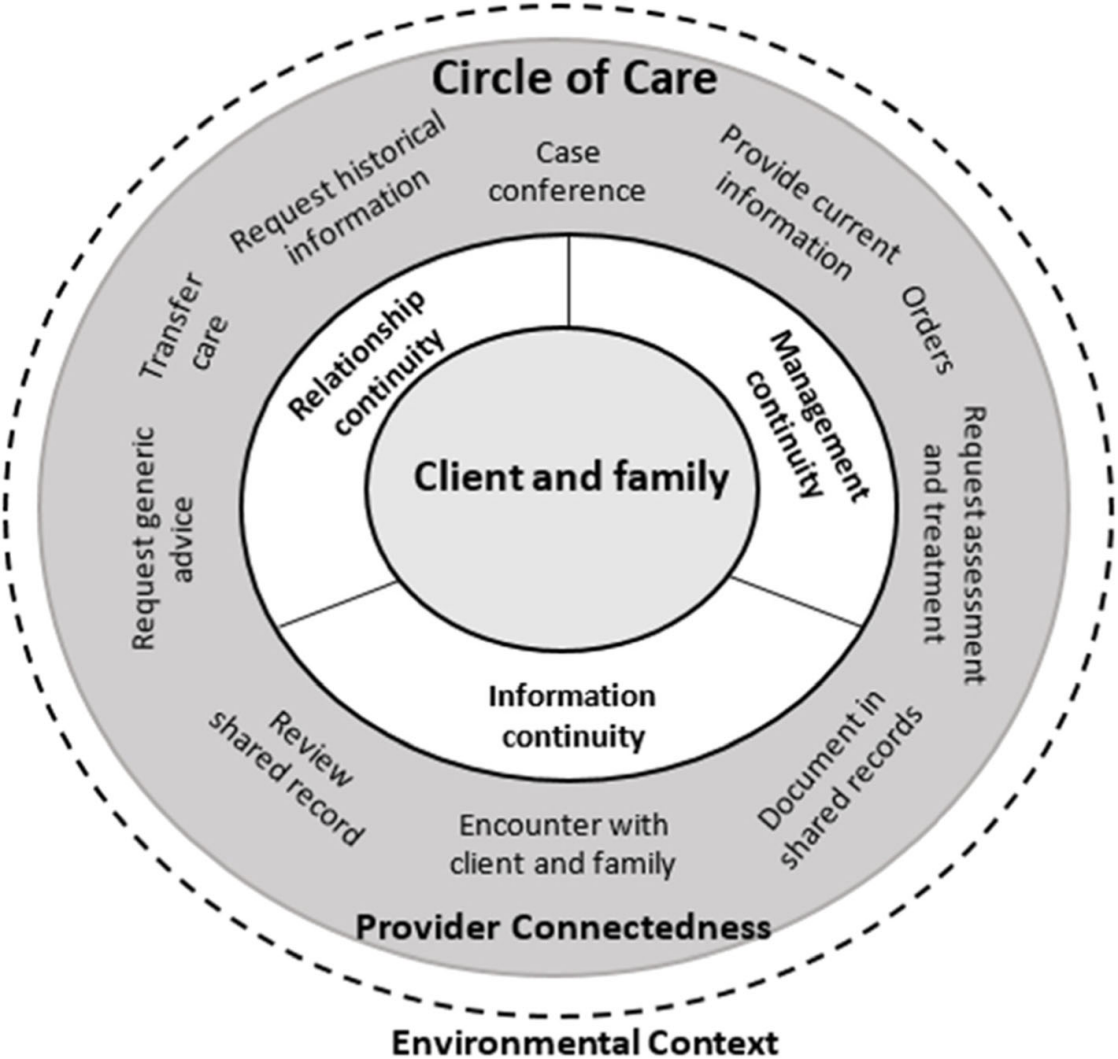
Extended continuity of care model by Price and Lau [15]

Interview recordings were analyzed using the software package ATLAS.ti for Windows [26]. To ensure consistency of coding, the first author developed a coding scheme based on the extended continuity of care model of Price and Lau (Fig. 2 and Table 2) [15]. The first and second author read each transcript independently. The first author selected text fragments from the transcripts. The second author read the transcripts and selected fragments and indicated to the first author if fragments were irrelevant or overlooked. Fragments were removed and/or added based on consultation between the first and second author.

**Table 2:** Extended continuity of care model [15]

Elements		Description
Original model of continuity by Haggerty and Reid [16]		
1. Client and family		The perceived coordination of care for a single client over time.
2. Information continuity		The capacity of information belonging to a client, between providers and over time, to facilitate continuity of care experience [27].
3. Management continuity		The extent to which services delivered by different healthcare professionals are timely and complementary such that care is experienced as continuous [28].
4. Relationship continuity		A sustained relationship between a patient and a specific healthcare professional [29].
Extended features of model of continuity by Price and Lau [15]		
1. Circle of care		An individual client’s healthcare system. The circle of care is a soft system that consists of the client, providers, other agents, and the information repositories (paper and electronic) related to that client. It is self-organizing, can span organizations, and changes based on the needs of the client and availability of resources [15].
2. Environmental influences		Factors outside the circle of care that can influence continuity of care – factors that are not related to the client, professionals, or information repositories.
3. Provider connectedness		Cohesiveness of the relationships between professionals in a circle of care.
4. Communication patterns	Transfer care	Handing off care responsibilities between care professionals of a similar capability.
	Provide current information	Ensuring that other professionals are aware of current findings and plans by sending information directly to named members of the circle of care.
	Encounter with client and family	Communicating with the client to examine the client’s condition share information, educate, and to develop a common understanding or plan.
	Request historical information	Seeking additional information from a particular professional, care team, or organization.
	Document in shared record	Documenting findings/plans in a location that is accessible to others (who have access).
	Review shared record	Review information shared by other members of the circle of care to increase knowledge of a client’s condition.
	Request generic advice	Request information and advice about options related to a client case.
	Request assessment and treatment	Contact another professional to request an action to assess and provide treatment recommendations to a client, based on their assessment.
	Orders	Request that specific activity be delegated to or performed by another professional.
	Case conference	To review, in real time with more than two individuals, the status and plans for the clients from multiple viewpoints.

All participants made statements about transfers of care, resulting in 701 fragments to be analyzed (mean 52.8 fragments per respondent, in a range from 25 to 87 fragments). At random, 10% of the fragments were selected to measure the inter-rater reliability. Statements about transfers of care were encoded according to the elements mentioned in the extended continuity of care model of Price and Lau [15]. The first and second authors analyzed the 10% of the fragments independently, using the coding scheme. The agreement on the elements between the two encoders was high, with Cohen’s kappa at .85. Differences in classification were discussed between the two assessors until consensus was reached. Consequently, the analysis of the remaining 631 fragments was divided between the two encoders. Factors that according to the respondents influence the continuity of care facilitating or impeding were indicated as such by the respondents themselves.

## Results

All 15 participants mentioned each element of the extended continuity of care model, except the circle of care. No additional elements were identified to supplement the continuity of care model. In the following paragraphs healthcare professionals’ perceptions of the relevant factors that influence continuity of care relating to the elements of the extended continuity of care model will be discussed, sorted by the number of fragments.

### Environmental influences

Environmental influences refer to factors outside the circle of care that impact continuity of care – factors not related to the client, professionals, or information repositories. The 15 participants mentioned environmental influences in 86 fragments. The participants mentioned one facilitating environmental factor for continuity of care, the usage of protocols and four impeding environmental factors for continuity of care, multiple locations, acute situations and high workload, language barriers, and night shifts.

#### Facilitating factors

An environmental factor facilitating continuity of care, mentioned by four participants, is following additional education on how to transfer clients and collaborate as a team in acute situations. Protocols that describe this are already in use and workshops are provided regularly, but participants mentioned that it is important that all professionals learn how to work with the protocols.

A resident obstetrician explains how his team uses a protocol after following a workshop:

*“The clinical midwives recently had training which included workshops to improve collaboration between themselves and to improve communication. An SBAR (situation, background, assessment, recommendation) workshop was also held to practice this. Nowadays in the delivery rooms SBAR guidelines specifically for obstetrics are available on notepads.”(C1)*

#### Impeding factors

Three participants mentioned that some healthcare organizations have multiple locations (for example, youth healthcare clinics are situated in several neighborhoods of a city). It is therefore difficult to find the professional that will take over healthcare provision.

A maternity care nurse described this impeding factor:

*“She [community midwife] says on the phone: ‘Then you just go to the Well baby clinic.’ But I do not know that. I don’t know where the Well baby clinics are located in the neighborhoods. That is outside my catchment area. Do I first have to get a list with the locations of all the Well baby clinics? That’s impossible. That is too unclear.” (B1)*

Four participants mentioned that due to acute situations and high workload, transferring care can be difficult. Distracted professionals during a transfer are undesirable, illustrated by this community midwife:

*“I think that work pressure on the other end of the phone is not good – hastiness is not good. If I have an emergency and I call for something, I can hear whether the transfer is acute or not. Especially if I’m thinking I have to consult, you can see that the person on the other end of the phone is distracted, which is not good.” (A4)*

A clinical midwife also mentioned a language barrier between her and resident obstetricians who originate from Flanders, the northern part of Belgium. Flemish is a variety of the Dutch language. This barrier was especially noticeable for her in acute situations, because during those moments communication needs to be quick and clear.

*“We have a lot of Flemish resident obstetricians here and sometimes you cannot understand them. That is really the language. They use different words. For example, you say: ‘About who are we talking now? Can you spell the name?’. Then you repeat it five times and they still do not know who you are talking about. Literal language barrier.” (A4)*

A community midwife and a maternity care assistant mentioned how working night shifts can impede the continuity of care.

*“Especially at night, if you are drowsy. Then you have to call someone and wake them up, and that person is not immediately ready to listen attentively [transfer at night].” (A2)*

### Provider connectedness

Provider connectedness describes the cohesiveness of the relationships between professionals in a circle of care. In 78 fragments, all 15 healthcare professionals mentioned their relationship with other members of the circle of obstetric and neonatal healthcare. They mentioned three facilitating factors (working together as a team, feeling connected/having mutual trust, and knowing colleagues in person) and two impeding factors (presence of hierarchy and not knowing who to contact).

#### Facilitating factors

The provider connectedness factor facilitating continuity of care, mentioned by four participants, was taking care of a client together, as a team. This was illustrated by a maternity care nurse:

*“You notice people thinking, ‘you are really a team,’ that they know exactly what the other one brings.” (B3)*

Seven participants talked about feeling connected to other professionals and how mutual trust is extremely important in working together as a team. Six participants expressed that knowing the other professionals in person facilitates care, especially when they work with the same people more often and in the same surroundings. One of the youth healthcare nurses explained that, because she knows the other professionals well, she contacts them more easily.

The following quotes by a maternity care assistant and a youth healthcare nurse depict these facilitating factors:

*“No, it is really a strong collaboration. You know each other personally, you trust each other because you work together so often. The midwife knows what you bring.”(B1)*

*“If you know someone, their name and face, and you also know how to find them easily, of course the connection is easily made. Then you will seek contact a lot sooner.” (D1)*

#### Impeding factors

A provider connectedness factor that four participants identified as impeding was the hierarchical levels between healthcare professionals. A perceived hierarchical difference between professions prevented a maternity care assistant from contacting another professional for additional information.

*“But who was present [childbirth], who do I need. I need to know exactly who was there at the time. Yes, but I am not going to speak to that person on the phone. The obstetrician was supervising, I will not get that person on the phone at all. Let it go.” (B2)*

Four participants mentioned another factor that may impede continuity of care – not knowing who to contact when asking for additional information. It takes more time to find out who the right person is to answer their questions.

*“And then she says: ‘Well, I can ask a colleague if she can call you back.’ But then I think: ‘Then it will go through another person ─ again.’ And then I do not know for sure if it is at the place where it should be. I want that certainty. Because if I have serious concerns, I think that I am obliged to know if my concerns end up with the right person.” (B1)*

##### Communication patterns

Price and Lau themed 10 communication patterns that were seen between care professionals, between healthcare organizations and between professionals and clients. The pattern “transfer of care” was mentioned often, in 193 fragments, which is why we will elaborate on this pattern in a separate paragraph. The same is done for the frequently mentioned patterns “Provide information” (93 fragments) and “Communicate with client and family” (87 fragments). The remaining seven communication patterns will be discussed in one paragraph, due to the lower numbers of fragments.

### Transfer of care

The pattern “Transfer of care” entails handing off care responsibilities between care professionals of a similar capability. All 15 participants, in 193 fragments, mentioned how they transfer care to other professionals. Four participants explained two facilitating factors (warm transfer and tools to make adequate choices) and one impeding factor (insufficient knowledge of protocols). Most statements about transfers of care explained between which healthcare professionals their clients are transferred. Three participants were more specific and explained their scope of practice – when their care provision ends and another healthcare professional takes over. Two participants mentioned that they let go of that client as soon as the transfer takes place. Other participants mentioned that they keep track of former clients and ask for feedback.

#### Facilitating factors

Participants described their views on how cold transfers and warm transfers influence the continuity of care for their clients. A cold transfer happens when a client is transferred on paper (for instance sending a letter), by telephone or via email. The healthcare professionals don’t meet each other in person to hand over the care. The reason for transferring the client is mentioned in a letter or email, or during a telephone call. This method is described as easy and time-saving. The opposite of this is the warm transfer, where the current and new healthcare professional meet each other to transfer their mutual client. The transfer reason is exchanged during the conversation and backed up with the client’s files. Four participants explained why they find warm transfers more facilitating for continuity of care, compared to cold transfers.

A maternity care assistant described why she favors a warm transfer for herself, but also for her clients:

*“Yes, absolutely [warm transfer is better than cold]. You write it piece by piece; if you do it verbally, you can explain a bit more than just on paper. It will also be remembered better because you are communicating it verbally.” (B3)*

*“What I experience with clients when I do a warm transfer, is that people find it less disturbing. At that moment, both maternity care nurses are there – one says goodbye and the other one becomes acquainted with the client. I could tell that the client was thinking: ‘Oh nice, I do not have to say anything, because it has already been arranged. It feels so familiar that I did not even notice the transfer at all. It is continuous.’” (B3)*

According to 10 participants, protocols and care pathways are tools to make adequate choices when transferring clients. The protocol those 10 participants mentioned most often is the SBAR method (situation, background, assessment, recommendation). This method is used to bring structure to any form of communication between healthcare professionals, especially in acute situations that require immediate attention and action.

A community midwife explained how using the SBAR method helped her with transferring clients:

*“If I follow that structure [SBAR], the transfer will go more smoothly. In another manner, the information will also be transferred, but this is a bit neater.” (A2)*

The SBAR method gives the healthcare professionals tools to communicate clearly with each other.

#### Impeding factor

Four participants mentioned as an impeding factor that the execution of the protocol does not always go smoothly. According to a community midwife, this is due to insufficient training possibilities with all members of the circle of care.

*“Yes, training with SBAR [room for improvement]. In city A there are joint training sessions and we do not have them here. We can participate in city A, but that is not convenient.”(A2)*

### Providing information

According to Price and Lau, providing information is about ensuring that other professionals are aware of current findings and plans by sending information directly to members of the circle of care [15].

In 93 fragments, all 15 participants shared several methods they use to transfer information. Nine participants also identified one facilitating factor (usage of standard formats) and one impeding factor (usage of different registration systems). Participants transfer information by using letters, emails, telephone calls, faxing, meeting in person or letting clients transfer their own information.

#### Facilitating factor

Nine participants mentioned that they use standard formats to register information about their clients, which facilitates information transfer. Some use a paper register system and other digital systems to store information.

#### Impeding factor

An impeding factor four participants mentioned is the usage of different digital registration systems. At the moment, organizations in the circle of birth care purchase software where they register information about their clients. Transferred information can be incomplete – either the healthcare professional or the client needs to request the prior professional to transfer the missing information.

Four participants expressed their wishes for a uniform data registration system, such as these community midwives:

*“Yes, that we do not have a uniform data system [obstacle]. That would be cool. Then it was settled.” (A1)*

*“It would be ideal if we had one file, so that we really only have to send the referral letter, or a telephone call, like: ‘I want to send or transfer this woman for a consultation.’ Then they would click and see what we have already done. I do not know if I’m going to experience that.” (A2)*

Providing and registering information about clients is achieved in many ways, depending on the organization a professional works for. When information needs to be transferred to other professionals, there is no uniform method of sharing.

### Communicate with client and family

The pattern ‘Communicate with client and family’ explains that professionals communicate with clients to examine their condition, share information, educate, and develop a common understanding or plan. All 15 participants, in 87 fragments, gave examples of how they communicate with their clients. Eight participants pinpointed one facilitating factor (24-h service point of contact) and two impeding factors (communicating in acute situations and honoring privacy).

#### Facilitating factor

After transferring a client, one community midwife and three maternity care assistants always tell the client that they can contact a professional if they have any questions.

*“If there is something, you can always call. We try to do this in the practice, that people can call us without hesitation. It is a bit silly that they have to worry unnecessarily, while I can explain it in one phone call, or sometimes they say: ‘I want to hear the heartbeat.’”(A2)*

#### Impeding factors

Two community midwives told us that they find it hard to communicate an acute situation or a pregnancy complication to their clients. On the one hand they want to inform the client about her situation and the reason why she needs to be transferred to a hospital, but on the other hand they don’t want to frighten the client and cause extra stress.

As illustrated by the following quotes:

*“Yes, one should prepare them a little for what they can expect. Sometimes it happens really fast. I can imagine that, because at that moment you are feeling like you are losing control and because of that you can end up with negative feelings.”(A2)*

*“Then I say clearly that I am going to transfer her and that a group of doctors in white coats will be coming into the room. That is normal procedure and she should not be afraid of that.”(A2)*

Another impeding factor that six participants mentioned was honoring the privacy of their clients. Participants mentioned that if they want to consult another professional about a treatment plan, the client needs to give her permission. If this permission is not given, the professional is not permitted to discuss the client’s record with other healthcare professionals.

As to communicating with client and family, the participants think it is important that clients know that they can contact them at any given time. They find it hard that honoring the privacy of their clients can impede the continuity of care, if they want to consult other colleagues.

##### Remaining components

The participants mentioned the seven remaining communication patterns much less often (Request historical information; Document in shared records; Review shared record; Request generic advice; Request assessment and treatment; Orders; Case conference). Although voiced in the interviews, the participants often did not mention facilitating or impeding factors for these patterns; they only illustrated their activities.

The only notable facilitating factor for continuity of care that community midwives, three secondary healthcare professionals and two youth healthcare nurses all shared was the importance of attending multidisciplinary meetings. During these meetings, multiple healthcare professionals discuss the situation and care plan for particular clients that need to be monitored (for example complications with the pregnancy/labor/maternity period or a complicated home situation).

A youth healthcare nurse shares her experiences with these meetings:

*“You can easily contact them and say something like, ‘What do you know about that family, what can I do, what is being done? Should we perhaps plan an MDG [multidisciplinary group meeting] to discuss with all professionals how to gain insight into what we can do?’ This way creates contact more easily. And I must say, it works very well.” (D1)*

## Discussion

Through using a vignette that described the care process of a pregnant woman and semi-structured interview questions, we studied how healthcare professionals transfer their clients, aim at continuity of care, and which factors influence the continuity of care. Participants mentioned nine factors that facilitate continuity of care: the usage of protocols, working together as a team, feeling connected/having mutual trust, knowing colleagues in person, warm transfer, tools to make adequate choices, using standard formats, 24-h service point of contact and attending multidisciplinary meetings. They also mentioned 10 factors that impede continuity of care: multiple locations, acute situations and high workload, language barriers, night shifts, different hierarchical levels, not knowing who to contact, insufficient knowledge of protocols, usage of different registration systems, communicating in acute situations and honoring privacy.

The extended continuity of care model by Price and Lau, which consists of four elements (circle of care, environmental influences, provider connectedness, and communication patterns) of which the latter consists of 10 patterns, was used in this study to investigate which factors facilitate and impede the continuity of care in the Dutch obstetric and neonatal healthcare system [15]. During the interviews, participants mentioned the three elements ‘environmental influences, ‘provider connectedness’, and ‘communication patterns’. The participants did not (or hardly) mention one element and some communication patterns of the Price and Lau model, namely ‘circle of care’ and the communication patterns ‘request historical information’, ‘document in shared record’, ‘review shared record’, ‘request advice’, ‘request assessment/treatment’, and ‘order’.

### Circle of care

Our analysis did not result in any fragments with facilitating and/or impeding factors about the circle of care (individual client’s healthcare system). An explanation may be that ‘circle of care’ is an overarching feature of the model and text fragments fitted better in other sub-elements of the model. However, it is obvious that many of the factors mentioned earlier (e.g. communication, trust, knowing each other in person, collaborating as a team) facilitate a good functioning of the 'circle of care'.

### Environmental influences

This element refers to factors outside the circle of care that influence continuity of care. Our participants mentioned multiple factors not related to the client, professionals, or information repositories. ‘Acute situations’ was a factor that stood out for us because, unlike regular transfers, acute transfers of care require different proceedings from healthcare providers.

Our participants fear that information may get “lost in translation” during an acute transfer. This fear increases when the acute situation happens at night and the professional already has a high workload. Wiegers and De Borst [8] showed in their research that professionals usually know how to contact other professionals in acute situations, but they are not always aware of the current protocols and agreements [8]. Especially in these acute cases, it is of the utmost importance that professionals communicate well with each other [20]. Acute transfers between professionals, especially during childbirth, are risk factors for a negative birth experience [12, 18]. Women who are giving birth and experience an acute transfer can have feelings of uncertainty about what is happening to them and their babies, where they are going and who is taking care of them [12]. Therefore, good communication with the client and family is just as important as proper communication between professionals for the continuity of care.

Gardner et al. [27], researched the influence of strategies such as case conferencing which can improve the relationships between healthcare professionals [27]. A systematic review by Powel et al [30], found that the implementation of such strategies could improve client satisfaction and health outcomes [30]. Patients with chronic disorders such as depression and diabetes showed improved health outcomes when they were treated by healthcare professionals that use case conferencing, protocols or agreements [31]. Unfortunately, in acute situations there is little room for case conferencing and other communication strategies need to be used.

The most impeding factors were found in the environmental influences, which shows that the participants are hindered in their work by external influences. Unfortunately, acute situations, multiple locations and night shifts, which were classified as impeding factors to continuity of care, will always be present. Professionals and management of healthcare organizations should implement communication strategies that are appropriate and effective for acute situations.

### Provider connectedness

This element describes the cohesiveness of the relationships between professionals in a circle of care. All participants in our study mentioned relationships with colleagues in the Dutch obstetric and neonatal healthcare system and the ability of such relationships to influence the continuity of care. Most facilitating factors were found in the provider connectedness, which highlights the importance of good cohesiveness of the relationships between professionals. Continuity of care appears to go more smoothly if the healthcare professionals feel connected to each other ─ if they function as a team, experience mutual trust, and know each other in person.

Multiple studies have concluded that it is of vital importance for professionals who are involved in transfers to know and trust each other, which not only benefits the provider-connectedness but also the experiences of the clients regarding the care provision [8, 17]. With these abilities, professionals are able to give adequate responses to each other [8].

Our participants mentioned two examples about how to start knowing colleagues in person: participating in combined courses for all professionals in the obstetric and neonatal healthcare chain aimed at improving continuity of care by using protocols, and participating in multidisciplinary group meetings. Wiegers and De Borst [8] also concluded that multidisciplinary group meetings improve communication and cooperation between members [8]. According to our participants, continuity of care can be compromised if some professionals in the healthcare system are not aware of the protocols.

Hierarchical differences between professionals in the healthcare system are perceived as impeding for provider connectedness and impact continuity of care. Colvin et al. [32], supported this view, having stated that a hierarchical relationship is a barrier for positive collaboration in an obstetric and neonatal healthcare system [32]. An obstetric and neonatal healthcare system with professionals who work together as a team without hierarchal differences therefore contributes to continuity of care.

### Communication patterns

The participants did not mention all communication patterns, but elaborated substantially on how they transfer their clients. This concerns two of the communication patterns – ‘transfer of care’ and ‘providing information’). All participants used standard forms to transfer clients. The forms are provided by the organizations they work for, but the instruments they use to transfer the content of the forms are different. While some participants use digital instruments (email or digital registration systems), others send the forms via regular post. Participants agreed that a uniform registration system to which all involved healthcare professionals have access would be ideal. This idea was also mentioned in a Dutch guideline from 2016 in which it is stated that the efficiency of healthcare provision would not only improve but would also prevent errors or interpretation mistakes when entering data [33].

All participants encouraged the usage of protocols, with SBAR the one that was mentioned most often, especially for acute transfers. Our participants were clear about their preferences for warm transfers (where clients are transferred face-to-face between colleagues, backed up by medical files) instead of cold transfers (where clients are transferred on paper, via telephone, email, or letter). No evidence was found to support conclusions about the effectiveness of transfer styles (warm versus cold transfers) for facilitating continuity of care [34, 35]. It has been investigated that transfers where the client is present (bedside transfers) increase the involvement of clients regarding their treatment, which can improve client satisfaction and experiences, and manage expectations better [36].

The communication pattern ‘communicating with client and family’ explains that professionals communicate with clients about their condition, they share information, educate, and develop a common understanding or plan. Our participants explained that by being transparent about possible complications that may affect their clients and by taking the time to explain their actions, they manage clients’ expectations. They don’t find this easy to do, because they don’t want to frighten their clients or worry them unnecessarily.

Other measures our participants took to reassure their clients is by explaining that healthcare professionals are accessible 24 h a day. In some cases, participants wanted to exchange client information with other healthcare professionals for feedback or advice. Privacy regulations were mentioned as an impeding factor in this process. However, with the client’s consent, the situation may be discussed between involved healthcare professionals.

The participants involved in our study mentioned that continuity of care benefits from a situation where communication between healthcare professionals and between healthcare professionals and their clients is optimal. The relationship between professionals is strengthened when the professionals regularly work which each other and know each other personally.

### Strengths and limitations

A strength of this study is that the experiences of almost all involved professional groups in the Dutch obstetric and neonatal healthcare system could be analyzed. As such, the study provides a sample picture of professionals’ experiences with factors that influence the continuity of care. As such, this manuscript adds to our knowledge of the facilitating and enabling factors of continuity of care mentioned by obstetric and neonatal healthcare professionals by using the extended model of continuity of care. Previously, this was only explored for palliative healthcare and primary healthcare in general.

One limitation of the project is that a vignette study does not monitor real proceedings regarding the transfer of a client, but only portrays the perspectives of healthcare providers. The answers given during the interview are therefore vulnerable to social desirability bias. However, questions about the real proceedings professionals undertake when they transfer their clients were included in the semi-structured interview. Another limitation is that more professionals who find transfers of care interesting or are highly involved with client transfers may have agreed to participate in this study than professionals who transfer clients less often. Non-responders are probably less interested in properly transferring their clients, which may have resulted in a too favorable or unfavorable picture of healthcare providers’ experiences. Finally, the non-iterative approach which was used in the procedures of this research could have resulted in not detecting new elements of the extended continuity of care model or not thoroughly examining the existing elements of the extended continuity of care model. Therefore a more structured topic guide or an iterative approach to the interviews with adaptation of the topic guide in response to earlier interview results could have given more answers related to some elements or new elements of the extended continuity of care model.

### Recommendations

We would advise obstetric and neonatal healthcare organizations to invest resources in providing regular multidisciplinary training sessions on transferring clients by using protocols, especially in acute situations. This factor was mentioned multiple times as a facilitating environmental influence and should therefore be implemented as a regular policy measure among obstetric and neonatal healthcare organizations. As a result, professionals will maintain their skills on how to efficiently transfer clients, and they will get to know the other professionals in person. Being familiar with other professionals in person was mentioned by multiple participants as an important facilitator for provider connectedness.

A uniform digital registration system for all involved obstetric and neonatal healthcare providers could be beneficial. The development and implementation of such a system was also mentioned as a facilitating communication pattern (providing information), and the lack thereof as impeding. Due to privacy and security concerns, a digital registration system is not yet available. However, because of the upcoming integrated Dutch obstetric and neonatal healthcare system, which entails close collaboration between the involved professionals, the chances of developing such a system have increased.

## Conclusions

We found a broad variety of factors facilitating and impeding continuity of care. In order to determine which factors should be included in the development of new protocols and agreements on transfers, it is important to know which factors have the most impact. Future research should investigate which factors significantly predict the continuity or discontinuity of care, using quantitative research methods.

## Supplementary information

**Additional file 1.** Interview protocol professionals. Interview protocol used to interview the healthcare professionals.

## Data Availability

The interview transcripts are not publicly available due to confidentiality restrictions, but are available from the corresponding author on reasonable request. The transcripts will be kept for 15 years at the Faculty of Behavioural, Management and Social Sciences, University of Twente, Enschede, The Netherlands.

## Abbreviations

MDG: multidisciplinary group meeting
SBAR method: situation, background, assessment and recommendation method
