# Supplementary material for: Through the professional’s eyes: transfers of care during pregnancy, childbirth and the postpartum period
Source: BMC Health Serv Res. 2020 Feb 11;20:108. doi: 10.1186/s12913-020-4941-0 (PMC7014703; doi:10.1186/s12913-020-4941-0)
Supplement: Supplementary file 1 — Additional file 1. Interview protocol professionals. Interview protocol used to interview the healthcare professionals. [file 12913_2020_4941_MOESM1_ESM.docx]

**Interview protocol professionals**

**Interview guide**

**(A) General information**

1. Are you a man or a woman?

- Man
- Woman

1. What is your age?

………… years old

1. What is your position in your organization?

………………………………………………………………………………………………………………………………………..

1. Can you describe your position?

………………………………………………………………………………………………………………………………………..

1. How many years have your worked for this organization?

……………………………………………………………………………………………………………………………………years

1. The subject of this study is transferring clients between healthcare providers. How would you describe the term ‘transfer of care’?

………………………………………………………………………………………………………………………………………..

1. Can you share with us the importance of a good transfer in your daily work?

………………………………………………………………………………………………………………………………………..

**(b) Scenario (see next page for illustration)**

We provide you with a fictional case. Please answer the questions as if the persona is your own client. Mrs. Visser is a woman of 30 years old. She has an intermediate vocational education, is of Dutch origin and married. Mrs. Visser is in good health and hereditary conditions are not present in her family. This is her first pregnancy. She lives in a medium-sized town with her husband.

**Scenario for community midwife**

Mrs. Visser wants to give birth at home. She discussed this with her community midwives and because there are no increased risks for complications, this is not a problem. During pregnancy, the community midwife estimates that the fetus is smaller than usual for its gestational age and decides to transfer the woman to the obstetrician for further evaluation. The obstetrician judges after multiple ultrasound examinations that the growth is adequate for the gestational age and that Mrs. Visser, as she wishes, can give birth at home.

Questions for community midwife

- How would you transfer Mrs. Visser to an obstetrician? (how to contact healthcare provider in secondary care?, how do you take into account the wishes of the client?)
- Do you receive feedback from the secondary healthcare professional? (how do they contact you?)

Around the due date, Mrs. Visser’s water breaks. Once labor starts, Mrs. Visser wishes pain relief. As a result, Mrs. Visser has to go the hospital for further guidance of her childbirth.

Questions for community midwife

- In the case of Mrs. Visser, what steps would you take in order to transfer Mrs. Visser to the hospital? (how to contact someone from secondary care?, do you stay with your (previous) client in the hospital?, how do you transfer information?)
- Does a transfer take place, from the obstetrician to you as a community midwife, after childbirth?
- Do you see Mrs. Visser after childbirth? (if so, how do you receive information about childbirth?)

**Scenario for resident obstetrician**

Mrs. Visser really wants to give birth at home. She has discussed this with her community midwives and because there are no increased risks for complications, this is not a problem. During pregnancy, the community midwife estimates that the fetus is smaller than usual for its gestational age and decides to transfer the woman to the obstetrician for further evaluation. The obstetrician judges after multiple ultrasound examinations that the growth is adequate for the gestational age and that Mrs. Visser, as she wishes, can give birth at home.

Questions for resident obstetrician

- How would Mrs. Visser be transferred from the community midwife to you? (type of contact, feedback?)
- What do you do if information about the client is missing? (do you contact the prior healthcare professional or ask Mrs. Visser?)
- How do you transfer Mrs. Visser to the community midwife after you decide that she can give birth at home?

Around the due date, Mrs. Visser’s water breaks. Once labor starts, Mrs. Visser wishes pain relief. As a result, Mrs. Visser has to go the hospital for further guidance of her childbirth. Mrs. Visser gives birth under supervision of a resident obstetrician. She receives pain relief. Childbirth has gone well and Mrs. Visser and her newborn child have to stay in the hospital a couple of hours for observation.

Questions for resident obstetrician

- How would Mrs. Visser be transferred from the community midwife to you? (type of contact, feedback)
- What do you do if information about the client is missing? (do you contact the prior healthcare professional or ask Mrs. Visser?)
- How do you transfer Mrs. Visser to the community midwife and/or maternity healthcare after childbirth?

**Scenario for maternity care assistant**

After childbirth, Mrs. Visser receives maternity healthcare for 8 days. The newborn is putting on weight, but is somewhat restless and cries continuously. The maternity care assistant observes some issues with Mrs. Visser, she is very tired and depressed.

Questions for maternity care assistant

- How is Mrs. Visser transferred to you after she gave birth in the hospital?
- What do you do if information is missing about the client or her newborn? (do you contact the previous healthcare professional or ask the client herself?)
- Who do you contact after observing the mentioned problems?
- How do you transfer Mrs. Visser to youth healthcare?

**Scenario for youth healthcare nurse**

In the second week after childbirth the well-baby clinic plans a home visit. A youth healthcare nurse visits the new family to introduce the healthcare organization. Everything goes well with the newborn and Mrs. Visser feels better too.

Questions for maternity care assistant

- How do you come into contact with a new client, such as Mrs. Visser and her newborn? (how is information transferred?)
- Do you have contact with previous healthcare professionals?
- What do you do if information about your client is missing?

**Illustration scenario**


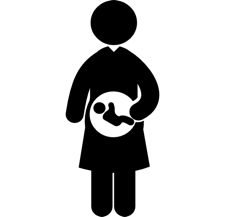


**A.** Mrs. Visser is pregnant for the first time. A community midwife guides the pregnancy.

A complication is noticed and Mrs. Visser is transferred to a hospital. After a while, the complications subside and Mrs. Visser is transferred back to the community midwife.


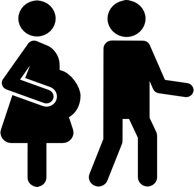


**B**. Mrs. Visser wants to give birth at home. During labor, Mrs. Visser decides she wants pain medication. Because of this, she needs to be transferred to a hospital.


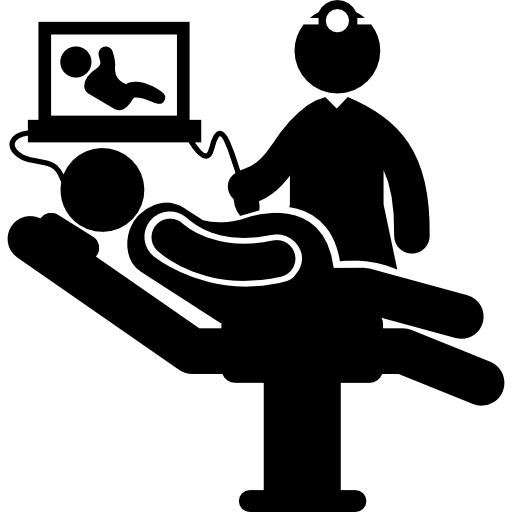


**C.** Childbirth is without problems, under the guidance of an obstetrician and a clinical midwife. After a few hours, Mrs. Visser is released from the hospital.


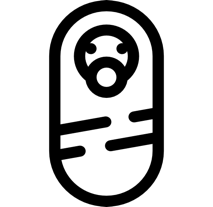


**D.** After childbirth, Mrs. Visser receives maternity care for eight days. The maternity care assistant notices that Mrs. Visser doesn’t sleep well, is very tired, and also depressed.


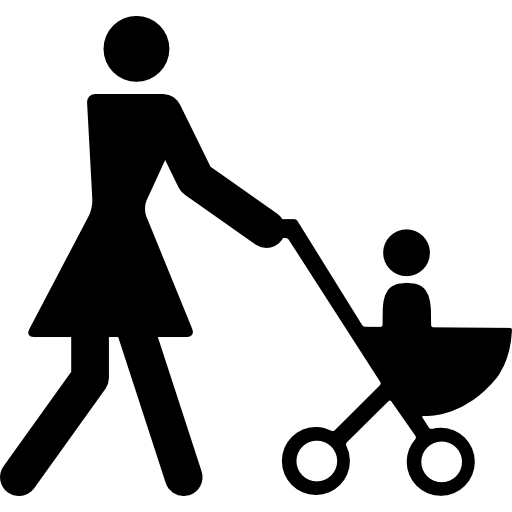


**E.** After two weeks, a youth healthcare nurse visits Mrs. Visser at home. Mother and baby are doing well.

**(C): questions on transfers of care**

1. On a scale of 1-10, how do you rate the quality of transfers that you receive?

………………………………………………………………………………………………………………………………………..

2. On a scale of 1-10, how do you rate the quality of transfers that you provide yourself?

………………………………………………………………………………………………………………………………………..

3. In your opinion, what is important for a good transfer / what are "good practices"?

………………………………………………………………………………………………………………………………………..

4. What can you do yourself to make the transfer as good as possible?

………………………………………………………………………………………………………………………………………..

5. Are there instances which you cannot influence regarding transfers of care?

………………………………………………………………………………………………………………………………………..

6. What problems do you experience when transferring clients? Is there room for improvement?

………………………………………………………………………………………………………………………………………..

Thank you for participating
